# Integration of Jasmonic Acid and Ethylene Into Auxin Signaling in Root Development

**DOI:** 10.3389/fpls.2020.00271

**Published:** 2020-03-10

**Authors:** Ping Xu, Ping-Xia Zhao, Xiao-Teng Cai, Jie-Li Mao, Zi-Qing Miao, Cheng-Bin Xiang

**Affiliations:** ^1^Basic Forestry and Proteomics Research Center, Fujian Agriculture and Forestry University, Fuzhou, China; ^2^School of Life Sciences and Division of Molecular and Cell Biophysics, Hefei National Science Center for Physical Sciences at the Microscale, University of Science and Technology of China, The Innovation Academy of Seed Design, Chinese Academy of Sciences, Hefei, China; ^3^Beijing Advanced Innovation Center for Tree Breeding by Molecular Design, Beijing University of Agriculture, Beijing, China

**Keywords:** *Arabidopsis thaliana*, root, auxin, jasmonic acid, ethylene, ethylene response factor, homeobox protein

## Abstract

As sessile organisms, plants must be highly adaptable to the changing environment by modifying their growth and development. Plants rely on their underground part, the root system, to absorb water and nutrients and to anchor to the ground. The root is a highly dynamic organ of indeterminate growth with new tissues produced by root stem cells. Plants have evolved unique molecular mechanisms to fine-tune root developmental processes, during which phytohormones play vital roles. These hormones often relay environmental signals to auxin signaling that ultimately directs root development programs. Therefore, the crosstalk among hormones is critical in the root development. In this review, we will focus on the recent progresses that jasmonic acid (JA) and ethylene signaling are integrated into auxin in regulating root development of *Arabidopsis thaliana* and discuss the key roles of transcription factors (TFs) ethylene response factors (ERFs) and homeobox proteins in the crosstalk.

## Introduction

Plant root systems represent the underground organs that provide mechanical support and uptake of nutrients and water. Depending on the species and environment, root systems show a high level of morphological diversity. Improved root architecture can increase the utilization of water and nutrients, which in turn helps increase crop yield. Most dicotyledons have tap root systems, while monocotyledons have fibrous root systems. The tap root system is composed of a developed primary root, lateral roots and adventitious roots, while the fibrous root system is mainly composed of adventitious roots (Martinez-de la Cruz et al., 2015; Zhang X. et al., 2019). The development of primary roots begins from embryonic development, whereas the lateral roots are initiated from asymmetrical divisions of the pericycle founder cell of primary roots. The root system morphology or architecture (RSA) is a highly plastic trait that is influenced by numerous biotic and abiotic factors (Osmont et al., 2007). An increasing number of studies in the model plant *Arabidopsis thaliana* have helped to address the underlying molecular mechanisms of this plasticity (Motte et al., 2019).

Root development occurs with the concerted action of multiple plant hormones (Petricka et al., 2012). Auxin has emerged as a core player on which other plant hormones integrate to regulate root development. Auxin synthesis, transport, and signaling pathways are important for plant root development. Indole-3-acetic acid (IAA) is the main naturally occurring auxin and the biosynthetic pathway of IAA has been clearly understood (Zhao, 2018). L-tryptophan is the major precursor of IAA synthesis, and the rate-limiting step of tryptophan synthesis is catalyzed by anthranilate synthase (a heterocomplex consisting of ASA1/2 and ASB1) (Sun et al., 2009; Casanova-Saez and Voss, 2019). ASA1 and ASB1 are also named WEI2 (Weak Ethylene Insensitive 2) and WEI7, respectively, since they were characterized from ethylene insensitive mutants of root growth (Stepanova et al., 2005). The two-step indole-3-pyruvate (IPA) pathway is the only IAA biosynthetic pathway that has been fully elucidated, and it is also the main pathway for IAA synthesis (Zhao, 2012). TAAs (Tryptophan Aminotransferase of Arabidopsis) and YUCCAs (YUCs) are enzymes that catalyze these two steps (Mashiguchi et al., 2011; Zhao, 2012). TAA1/WEI8, like ASA1 and ASB1, was also identified from the ethylene insensitive mutant *wei8* (Stepanova et al., 2008; Tao et al., 2008). Polar distribution is characteristic of auxin, which is mediated by PIN-FORMED (PIN) and AUXIN1/LIKE-AUX1 (AUX1/LAX) family members under strict regulations (Band et al., 2014; Adamowski and Friml, 2015). Localized auxin biosynthesis has been shown to play critical roles in root development as well (Zhao, 2010, 2018). Besides, the auxin signaling pathway is intensively studies recently with a focus on the fine regulation mechanisms of the IAA (INDOLEACETIC ACID-INDUCED PROTEIN) -ARF (AUXIN RESPONSE FACTOR) network (Wang and Estelle, 2014). Briefly, the auxin receptor TIR1/AFB (TRANSPORT INHIBITOR RESPONSE1/AUXIN SIGNALING F-BOX) binds to auxin causing degradation of IAA proteins that interact with ARF transcription factors (TFs) (Leyser, 2018; Gallei et al., 2019). ARFs bind to the auxin response elements (AREs) in promoters of target genes to regulate gene expression (Gallei et al., 2019).

During the stage of embryogenesis, auxin distribution patterns determine the position around which the embryonic roots start growing. In a later stage, auxin distribution patterns in and around the meristem determine root meristem activity and lateral root spacing (Motte et al., 2019). The development of lateral root is closely related to auxin, including its synthesis, transport and signal transduction (Osmont et al., 2007; Motte et al., 2019).

The gaseous phytohormone ethylene is well-known for its functions in plant maturation and senescence. In addition, numerous studies have shown that ethylene is involved in various plant growth and developmental processes, including root growth (Ruzicka et al., 2007; Swarup et al., 2007; Lewis et al., 2011; Street et al., 2015; Mao et al., 2016; Miao et al., 2018). The function of jasmonic acid (JA) in plant injury and defense responses has been thoroughly studied, and its roles in growth and development has also been widely reported (Kazan and Manners, 2013; Cai et al., 2014; Ye et al., 2019). Like other hormone signaling pathways, ethylene and JA signaling are integrated into auxin in root development, largely through TFs acting as the key crosstalk nodes.

### Ethylene-Auxin Crosstalk in Root Development

Ethylene is an important regulatory signal in regulating the process of root development (Ruzicka et al., 2007; Swarup et al., 2007; Street et al., 2015). Plants produce more ethylene when exposed to external stimuli (Wang et al., 2002). Ethylene binds to ETR1(ETHYLENE RESPONSE 1) receptor family on the endoplasmic reticulum (ER) membrane, leading to inactivation of the S/T protein kinase CTR1 (CONSTITUTIVE TRIPLE RESPONSE 1), which functions to repress EIN2 (ETHYLENE INSENSITIVE 2). After detaching from CTR1, EIN2 can be cleaved to release EIN2 C-terminal (EIN2C). The EINC has two levels of regulation of EBF1/2 (EIN3-BINDING F BOX PROTEIN 1/2). On the one hand, EIN2C binds to 3′-UTR of *EBF1/2* in the cytoplasm to inhibit its translation (Li et al., 2015; Merchante et al., 2015), and on the other hand, EIN2C is translocated into the nucleus to promote the degradation of EBF1/2 (Qiao et al., 2012; Dolgikh et al., 2019), both leading to stabilization of EIN3/EIL1 (ETHYLENE-INSENSITIVE3-LIKE1) to activate ethylene response genes. Although ethylene is best known for triggering fruit ripening, it also plays a crucial role in regulating root development. In response to ethylene or its precursor ACC (1-aminocyclopropane-1-carboxylic acid) treatment, the root of Arabidopsis seedlings shows three growth responses: rapid downregulation of cell elongation, increased root width, and induction of ectopic root hairs, which collectively will provide plants with greater anchorage and more dynamic regulation of root growth (Swarup et al., 2007).

Inhibition of root growth by ethylene depends on auxin biosynthesis, transport and signaling pathway (Ruzicka et al., 2007; Swarup et al., 2007). Ethylene up-regulates expression of auxin synthesis and transport-related genes in Arabidopsis roots, resulting in a high concentration of auxin that inhibits cell elongation (Ruzicka et al., 2007; Strader et al., 2010). Ethylene modulates the auxin transport machinery by directly or indirectly regulating the expression of auxin efflux (*PINs*) and influx (*AUX1*) carriers (Ruzicka et al., 2007). A subsequent study showed that ethylene can negatively regulate cell proliferation in addition to inhibiting cell elongation and SHY2 (SHORT HYPOCOTYL 2)/IAA3 mediated this effect in the root meristem (Street et al., 2015). It has been found that in Arabidopsis seedlings CTR1 transduces the ethylene signal to EIN2 in the root and then affects *PIN2* expression to modulate the root stem cell niche maintenance (Mendez-Bravo et al., 2019). The screening experiment on the ethylene overexpression mutant *eto1* identified a small molecule named L-kynurenine (Kyn), which could inhibit ethylene-directed auxin biosynthesis and root growth by inhibiting TAA1’s activity (He et al., 2011). *POLARIS* (*PLS*), encoding a predicted functional 36-amino acid peptide, is required in ethylene-mediated root inhibition through regulating auxin transport and affecting microtubule cytoskeleton dynamics (Chilley et al., 2006). The *PLS* expression is activated by auxin and suppressed by ethylene, and PLS peptide in turn negatively regulates the ethylene signaling pathway (Chilley et al., 2006). It was reported that ethylene can induce an oxidase named MINE, which produces pyridoxal-5′-phosphate (PNP), and PNP acts as a cofactor in TAA1/TAR-dependent auxin biosynthesis, which in turn influences ethylene-auxin crosstalk in Arabidopsis root (Kim et al., 2018).

Ethylene is involved in regulating the growth and development of not only primary roots but also lateral roots. Increased endogenous ethylene or ACC treatment activates *PIN3/7* expression thereby enhancing auxin transport and reducing lateral root formation (Lewis et al., 2011). Auxin signaling affects the cell division pattern of lateral root primordium by regulating the expression of the ERF (ethylene response factor) family transcription factor *PUCHI*, which is required for the proper pattern of early lateral root primordia (Hirota et al., 2007). PLS, the small peptide mentioned above, is also required in lateral roots initiation via ethylene-mediated auxin transport to the pericycle (Chilley et al., 2006).

Adventitious root initiation and development are also regulated by ethylene-auxin crosstalk. Ethylene was reported to inhibit adventitious rooting in Arabidopsis dark-grown seedlings by negatively regulating auxin biosynthesis (Veloccia et al., 2016). When applied together with IBA (indole-3-butyric acid), ethylene promotes the conversion of IBA to IAA and thus the development of adventitious roots (Veloccia et al., 2016). Ethylene-auxin crosstalk also regulates the initiation of adventitious roots near cut sites where the levels of auxin and ethylene both increase (Guan et al., 2019).

### JA-Auxin Crosstalk in Root Development

Jasmonates are well-known lipid-derived compounds as key regulators in plant growth and development as well as in plant stress responses. JA participates in the regulation of root growth, seedling development, flower development, root regeneration, seed development, seed germination, tuber formation and senescence (Wasternack and Hause, 2013; Ye et al., 2019; Zhang G. et al., 2019). JA regulates root growth in many aspects, including inhibition of primary root (Chen et al., 2011), promoting lateral roots formation (Cai et al., 2014), negatively regulating adventitious roots (Gutierrez et al., 2012; Lakehal et al., 2019), and inducing root regeneration (Ye et al., 2019; Zhang G. et al., 2019). Most of these processes are achieved via cross-talking with auxin.

Root growth inhibition is one of the first discovered features of JA. By screening mutants insensitive to JA-mediated root inhibition, a number of regulatory factors in the JA signaling pathway were revealed, such as JAR1 (JASMONATE RESISTANT 1) (Staswick et al., 1992), MYC2/JAI1 (JASMONATE INSENSITIVE 1) (Berger et al., 1996), and COI1 (CORONATINE INSENSITIVE 1) (Feys et al., 1994). JA inhibits root elongation by reducing both cell counts and cell dimension, suggesting that JA-induced primary root growth inhibition is a complicated process involving diverse cellular processes in different root tissues (Chen et al., 2011, 2012). JA-mediated inhibition of root development is auxin-dependent (Wasternack and Hause, 2013). JA activates *MYC2*, leading to the repression of *PLT1* (*PLETHORA1*) and *PLT2* in root stem cell niche (Chen et al., 2011). *PLTs* encodes members of the AP2/EREBP transcription factor family and are key effectors for the establishment of the stem cell niche during embryonic pattern formation. They respond to auxin accumulation and this response depends on auxin-responsive TFs. Therefore, PLTs serve as a key node for JA-auxin crosstalk in regulating the maintenance of the stem cell niche in roots (Chen et al., 2011).

Jasmonic acid is also involved in regulating lateral roots development. In response to methyl jasmonate (MeJA) treatment, Arabidopsis wild type produces more lateral roots, while the mutant *asa1-1* does not produce lateral roots (Sun et al., 2009). The JA receptor COI1 plays a critical role in the formation and even distribution of lateral roots (Raya-Gonzalez et al., 2012). In the *coi1-1* mutant, the lateral roots displayed uneven distribution and JA failed to induce more lateral roots (Raya-Gonzalez et al., 2012). In the root, MeJA activates the transcription of *ASA1* and several other auxin biosynthesis-related genes, such as *YUCCA2* (Cheng et al., 2006), *ASB1* (Stepanova et al., 2005), and *NITRILASE 3* (*NIT3*) (Kutz et al., 2002). JA failed to increase lateral root initiation in mutants with disrupted auxin signaling, like *slr1* (*iaa14*) and *arf7/19* double mutant (Sun et al., 2009), which further supports that JA-induced lateral root formation is auxin-dependent. Activated expression of the transcription regulator *HDG11* (*HOMEODOMAIN GLABROUS11*) increases the level of JA in the roots by directly up-regulating the expression of several genes encoding JA biosynthetic enzymes, resulting in enhanced auxin signaling and lateral root formation (Cai et al., 2015). MeJA can also induce *YUC8* and *YUC9* expression and thus participate in auxin-mediated primary root growth and lateral root initiation (Hentrich et al., 2013).

Jasmonic acid can exert negative effects on adventitious root formation (Gutierrez et al., 2012). ARF6, ARF8, and ARF17 act upstream of *Gretchen Hagen3.3 (GH3.3), GH3.5*, and *GH3.6*. These three GH3s inactive JA by conjugating JA to amino acids Asp, Met, and Trp, and therefore promote adventitious rooting (Gutierrez et al., 2012). The effect of JA in adventitious root development depends on experimental conditions. At low sub-micromolar concentrations, MeJA has been shown to promote adventitious root development when applied together with IBA, and this process does not involve regulation of ARF6 or ARF8 expression (Fattorini et al., 2018).

It is well known that plants can regenerate tissues and even complete organs after damage. Recently, Zhou et al. (2019) reported that the synergy between jasmonate and auxin signaling pathways promotes root regeneration by activating root stem cells (Zhou et al., 2019). In this process, JA induces *ERF109*, *CYCLIN D6;1* (*CYCD6;1*), and *ERF115* expression to activate stem cell and promote tissue regeneration. Auxin also activates key regeneration regulators of this pathway (Zhou et al., 2019).

### TFs Involved in JA-Auxin and Ethylene-Auxin Crosstalk

Transcription factors are specific implementers of numerous regulation processes. Each hormone crosstalk involves many important TFs. Here we focus on ERF family and HD-ZIP TFs in JA-auxin and ethylene-auxin crosstalk.

**FIGURE 1:**
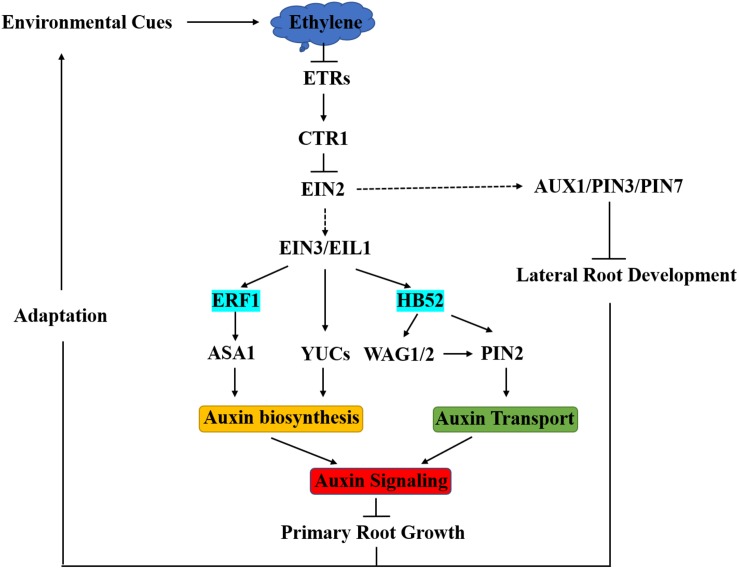
Integration of Ethylene into Auxin Signaling in Arabidopsis Root Development. Environmental cues trigger the biosynthesis of ethylene in Arabidopsis, and then ethylene binds to ETR receptors to inactivate CTR1, which functions to repress EIN2. When EIN2 is released by CTR1, it can be cleaved and then helps to stabilize EIN3/EIL1, leading to the activation of downstream transcriptional cascades. Ethylene inhibits primary root growth by regulating auxin biosynthesis, transport, and signaling. ERF1 and HB52 function as crosstalk nodes between ethylene and auxin in this process. An increase in endogenous ethylene enhances auxin transport and reduces lateral root formation depending on AUX1, PIN3, and PIN7. The ERF1 and HB52 regulatory modules are part of the molecular mechanisms in the adaptive response of root growth to environmental cues.

Ethylene response factor family TFs are plant specific and involve in a variety of plant development processes and stress responses. Many ERFs are responsive to ethylene. ERF1 negatively regulates primary root elongation in an auxin biosynthesis-dependent manner. Being downstream and a direct target of EIN3, ERF1 activates *ASA1* by binding to GCCGCC motifs (GCC-boxes) in the promoter of *ASA1*. The up-regulation of *ASA1* increases auxin biosynthesis, promotes auxin accumulation in root tip, and consequently suppresses root elongation (Mao et al., 2016). Therefore, ERF1 acts as a critical crosstalk joint connecting ethylene and auxin in regulating primary root elongation (Figure 1).

HOMEOBOX PROTEIN52 (HB52) belongs to the HD-ZIP transcription factor family. The HD-ZIP transcription factor family only found in plants with 47 members in Arabidopsis. According to protein structures and functions, HD-ZIP family members can be divided into four subfamilies (I-IV), and HB52 belongs to the HD-ZIP I subfamily (Ariel et al., 2007). HD-ZIP I family genes are responsive to external stimuli like drought, high temperature, osmotic stress, and lights. HB52 was identified from the study of ethylene-mediated root inhibition (Mao et al., 2016). *HB52* is highly expressed in roots and is responsive to the ACC treatment as a direct target of EIN3. HB52 regulates primary root elongation through affecting auxin transport. HB52 binds to the homeodomain-binding *cis*-elements in the promoters of *PIN2* and *WAG1/2* to activate their expression (Figure 1). WAG1/2, closely related to PINOID, can phosphorylate PIN2 to increase its auxin efflux carrier ability (Willige et al., 2012). Therefore, HB52 serves as another important crosstalk node between ethylene and auxin to regulate root elongation (Miao et al., 2018). Together, ERF1 and HB52 constitute the ethylene-responsive modules for auxin biosynthesis and transport, respectively, in root elongation regulation.

**FIGURE 2:**
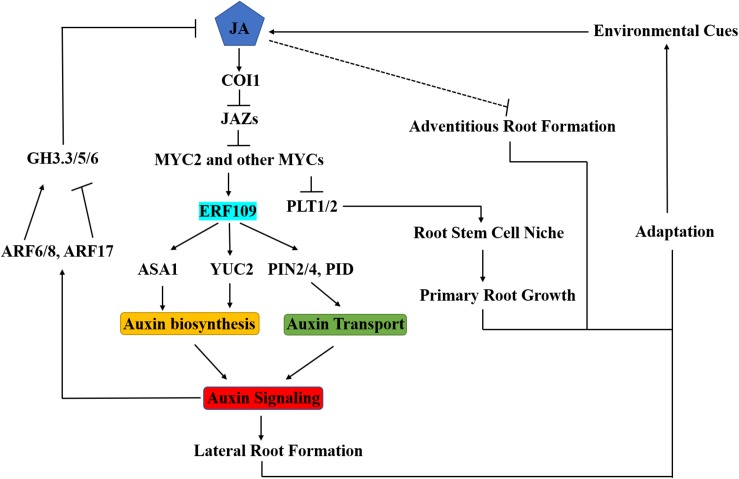
Integration of JA into Auxin Signaling in Arabidopsis Root Development. Plants generate JA in response to environmental cues. COI1 receptor perceives JA, and then recruits JAZs subjected to degradation. Subsequently, MYC2 can activate transcription of early JA-responsive genes. JA promotes lateral root formation by regulating auxin biosynthesis (via ASA1 and YUC2) and transport (via PID and PIN2/4). Transcription factor ERF109 functions as a key crosstalk node in this process. JA inhibits primary root development by repressing the expression of *PLT1* and *PLT2*. Auxin modulates JA homeostasis by regulating GH3.3/5/6 through ARF6/8/17, then influences adventitious root formation. Therefore, the ERF109 regulatory module plays critical roles in the growth and development of lateral, primary and adventitious roots in the adaptive response of the root system to environmental factors.

ERF109 is another member of the ERF family and responsive to the JA signaling pathway. ERF109 binds to GCC-boxes in the promoter regions of its target genes. Under normal conditions, *ERF109* is expressed at a very low level in roots. After MeJA treatment, the transcription level of *ERF109* was significantly induced in both roots and shoots, especially in the lateral root primordium region and the tip and base of lateral roots (Cai et al., 2014). Genetic analyses showed that ERF109 positively regulates lateral root formation through upregulating auxin biosynthesis. *In vitro* and *in vivo* experiments showed that ERF109 binds to the GCC-boxes in *ASA1* and *YUC2* promoters and directly activates their expression, leading to increased auxin biosynthesis and accumulation in the root (Cai et al., 2014). Thus, ERF109 serves as an important crosstalk node between JA and auxin signaling (Figure 2). Recently, three research groups independently reported that ERF109 has a novel function in plant regeneration depending on its roles in upregulating *ASA1* expression (Ye et al., 2019; Zhang G. et al., 2019) or activating *ERF115* and *CYCD6;1* (Zhou et al., 2019).

ERF1, ERF109, and HB52 are representative TFs involved in the crosstalk of JA-auxin and ethylene-auxin signaling pathways in regulating root development. Other TFs participated in the processes are yet to be identified.

## Conclusion and Perspectives

Crosstalk between hormone signaling are fundamental process in plant development, yet the underlying mechanisms are far from clear. In this review, we summarized recent advances on the understanding of ethylene and JA integration into auxin signaling in the regulation of root development.

Auxin plays the central role in regulating root development. Plant roots constantly perceive environmental cues and generate hormonal signals in order to adjust developmental programs for better adaptation to the changing surroundings. JA and ethylene are two representative hormones in plants responding to environmental changes. These two hormonal signals can be relayed to auxin signaling, the master regulator of root development. In the signal relay, TFs play critical roles to integrate other hormonal signal into auxin signaling through modulating auxin biosynthesis (for example, ERF1 and ERF109) or auxin transport (for example, HB52) to fine-tune the regulation of primary root growth and/or lateral root formation.

To unravel the complete network of JA-auxin and ethylene-auxin crosstalks in root development, we need to identify the more components involved in these processes, as well as understand the spatial-temporal relationships between these components. Some attempts have been recently made by identifying the root epidermis cells where the interaction between ethylene and auxin takes place (Vaseva et al., 2018; Mendez-Bravo et al., 2019). Moreover, local auxin biosynthesis is critical in ethylene-auxin crosstalk (Brumos et al., 2018). With the advance of new technology such as single-cell sequencing and high-resolution microscope, in-depth details in the crosstalk will be revealed.

## Author Contributions

PX and C-BX conceived the mini-review. PX wrote the mini-review. All authors contributed to the discussion and revision.

## Conflict of Interest

The authors declare that the research was conducted in the absence of any commercial or financial relationships that could be construed as a potential conflict of interest.
